# Enhancement of anisotropy energy of SmCo_5_ by ceasing the coupling at 2c sites in the crystal lattice with Cu substitution

**DOI:** 10.1038/s41598-021-89331-z

**Published:** 2021-05-12

**Authors:** Syed Kamran Haider, Hieu Minh Ngo, Dongsoo Kim, Young Soo Kang

**Affiliations:** 1 Department of Chemistry, Sogang University, 35, Baekbeomro, Mapogu, Seoul, 04107 South Korea; 2 Powder and Ceramics Division, Korea Institute of Materials Science, Changwon, Gyeongnam 51508 South Korea; 3 Convergence Research Center for Development of Mineral Resources, Korea Institute of Geoscience and Mineral Resources, Daejeon, 34132 South Korea

**Keywords:** Chemistry, Materials science, Nanoscience and technology

## Abstract

SmCo_5_ and SmCo_5−x_Cu_x_ magnetic particles were produced by co-precipitation followed by reduction diffusion. HRTEM confirmed the Cu substitution in the SmCo_5_ lattice. Non-magnetic Cu was substituted at “2c” site in the SmCo_5_ crystal lattice and effectively stopped the coupling in its surroundings. This decoupling effect decreased magnetic moment from SmCo_5_ (12.86 μ_B_) to SmCo_4_Cu (10.58 μ_B_) and SmCo_3_Cu_2_ (7.79 μ_B_) and enhanced anisotropy energy from SmCo_5_ (10.87 Mega erg/cm^3^) to SmCo_4_Cu (14.05 Mega erg/cm^3^) and SmCo_3_Cu_2_ (14.78 Mega erg/cm^3^). Enhancement of the anisotropy energy increased the coercivity as its values for SmCo_5_, SmCo_4_Cu and SmCo_3_Cu_2_ were recorded as 4.5, 5.97 and 6.99 kOe respectively. Being six times cheaper as compared to Co, substituted Cu reduced the price of SmCo_3_Cu_2_ up to 2%. Extra 15% Co was added which not only enhanced the M_r_ value but also reduced the 5% of the total cost because of additional weight added to the SmCo_3_Cu_2_. Method reported in this work is most energy efficient method on the synthesis of Sm–Co–Cu ternary alloys until now.

## Introduction

Among permanent magnet materials, SmCo_5_ constitute the second strongest class of magnets. With the K_u_ value larger than 1.19 × 10^8^ erg/cm^3^ SmCo_5_ alloy has a very high uni-axial magneto-crystalline anisotropy and the highest Curie temperature among permanent magnetic materials^1–7^. Because of high curie temperature they also have special applications, such as high-end engines in motorsports, where magnets are exposed to temperatures above 240 °C. Transition metals substitution for Co into SmCo_5_ can change the values of coercivity, M_r_ and M_s_^8–11^. It was theoretically predicted that by substitution of Cu with Co, coercivity of SmCo_5_ can be increased on the expense of M_r_ and M_s_ values^12^. Decrease in the M_r_ is the serious disadvantage that reduces the energy density/BH_max_ despite of enhancement of the coercivity.

Second disadvantage associated with SmCo_5_ magnets is their high cost. Sm is the most expensive element among its group and Co is also more expensive than Fe and these make the SmCo_5_ most expensive class of the permanent magnets. Biggest reason behind high cost of SmCo_5_ is the low quantity of the Sm and Co in the earth crust as shown in Fig. S-1-a. Cu is almost twice abundant (0.0068%) as compared to the Co (0.0003%) in the earth crust and this is one of the reasons that it is six times cheaper as compared to the Co (Fig. S-1-b). Another reason for the low price of Cu is the acquisition of Cu as the byproduct, during the extraction of Co from ores by pyro-metallurgy, hydro-metallurgy, and vapor-metallurgical methods^13^. Hence some ores those are primarily source of the Co (e.g. Carrollite) are also an important source of Cu.

Cu substitution for the Co can reduce the cost of most expensive magnet, SmCo_5_. But the price of SmCo_5_ is critically determined by the Sm due to the high price of Sm (750 USD/kg) as compared to the Co (28 USD/kg). Although Co is six times more expensive as compared to the Cu but Co substitution with Cu (in SmCo_3_Cu_2_) decreases the price of the magnet produced, only up to 2%. Another important factor that may reduce the price of the SmCo_3_Cu_2_ is use of extra fused Co phase. This extra Co not only enhances the magnetic properties but also increases the overall weight of the produced SmCo_3_Cu_2_. SmCo_5_ consists of almost 33% of Sm by weight, hence 15% addition of Co saved the 5% of the Sm. By considering both the factors (substitution of Co with Cu and addition of extra Co), overall price of SmCo_3_Cu_2_ could be reduced up to 6%. Reduced price of the SmCo_5_ in this work may have a large impact, because global market value of the SmCo_5_ magnet produced per year is approaching worth 1 billion USD in near future (Fig. S-1-c).

Third problem with the synthesis of Sm–Co–Cu ternary alloys is the high consumption of energy. Te´llez-Blanco et al.^14,15^ annealed Sm, Co and Cu for 504 h (21 days) at 1000 °C. Nishida et al. performed the annealing at more than 800 °C for 160 h^16^. Gabay et al. annealed the Sm, Co and Cu for 100 h (50 h at 1050 °C then 50 h 350–450 °C)^17^. Researchers those did not use simple annealing, used expensive methods like sputtering, arc melting, electron beam heating and Induction melting. Even after induction melting at 1430 °C, Suresh et al. later annealed the sample at 900 °C for 4 h for homogenization^18^. Annealing at such high temperature also leads to the loss of Sm which is evaporated and compensated with the addition of extra Sm. In this work reduction diffusion method is used for the synthesis of the magnetic particles which energy and time efficient as compared to the regular physical methods^19–22^.

This study suggests an eco-friendly chemical method for the synthesis of the Cu substituted SmCo_5_ particles. In this energy efficient process, precursors were annealed at 900 °C for only 2 h. Cu substitution enhanced the coercivity, while reduction in the Mr value caused by Cu substitution is compensated by addition of extra Co. Cost of the SmCo_5_ is reduced by substitution of Cu with Co. Extra added Co also increased the weight of SmCo_5−x_Cu_x_ magnet which further reduced its cost.

## Experimental section

### Materials

Samarium chloride (SmCl_3_^.^6H_2_O, 99% purity), cobalt chloride (CoCl_2_^.^62H_2_O), copper chloride (CuCl_2_^.^2H_2_O, 99% purity), and potassium chloride (KCl, 99% purity) were obtained from Sigma Aldrich. 16 mesh granular Ca (99.5% purity) was obtained from alfa Aesar. These reagents were used without further purification. Milli-Q IQ 7000 water purifying system was used to obtain deionized water.

### Preparation of SmCo_5_ and SmCo_5−x_ Cu_x_ particles

In the first step, chloride precursors were changed to hydroxides by the co-precipitation method. Chloride compounds of Sm, and Co and Cu were dissolved in DI water. SmCl_3_^.^6H_2_O, CoCl_2_^.^6H_2_O and CuCl_2_^.^2H_2_O were used as precursors and their molar masses were noted as 364.8, 237.95 and 170.48 g/mol respectively. In order to prepare SmCo_5_, Sm:Co molar ratio was kept as 1:4.2. Hence, 2.379 g of CoCl_2_^.^6H_2_O, and 0.870 g of SmCl_3_^.^6H_2_O were dissolved in 100 ml of de-ionized water. Extra Sm precursor was used because some Sm is evaporated during the reduction-diffusion process.

For synthesis of SmCo_4_Cu and SmCo_3_Cu_2,_ Sm:Co:Cu molar ratios were kept as 1:3.36:0.84 and 1:2.52:1.68 respectively. 15% extra Co was further added to the SmCo_5−x_Cu_x_ that changed the Sm:Co:Cu molar ratio. Hence, for SmCo_4_Cu Molar ratio of Sm:Co:Cu was taken as 1:3.86:0.86. Meanwhile for SmCo_3_Cu_2_ Molar ratio of Sm:Co:Cu was taken as 1:2.90:1.68.

Experimentally for synthesis of SmCo_4_Cu, SmCl_3_^.^6H_2_O (0.870 g), CoCl_2_^.^6H_2_O (2.186 g) and CuCl_2_^.^2H_2_O (0.341 g) were dissolved in 100 ml of water. Meanwhile for synthesis of SmCo_3_Cu_2_, SmCl_3_^.^6H_2_O (0.870 g), CoCl_2_^.^6H_2_O (1.643 g) and CuCl_2_^.^2H_2_O (0.682 g) was dissolved in 100 ml of water.

A 3.5 M NaOH solution was added drop-by-drop to raise the pH of the solution up to 13, so that all of the metal chlorides could be changed to metal hydroxide. The reaction mixture was stirred continuously during the addition of NaOH solution. While maintaining the pH at 13, the solution was stirred for 1 h. Then, products were washed twice with DI water and ethanol, and dried at 80 °C for 1 h. XRD, TEM, and TEM-EDS analysis of SmCoCu hydroxides are provided as Fig S-2, S-3 and S-4 and S-5 in supporting information. The second step was the reduction-diffusion reaction. Hydroxides obtained from the first step were mixed with Ca and KCl in a glove box and then pressed into pellet form. Weight ratio of Ca, hydroxides and KCl was kept as 6:1:1. The pellet was reduced and diffused in a tube furnace by heating at 900 °C for 2 h, while Ar was flowing in the furnace. CaO produced during R-D can reduce the magnetic properties^23,24^. Hence, the product was washed with water again and again to remove CaO completely and washed twice with acetone before being stored in inert conditions. All steps of the experimental procedures are shown in Fig. S-6. Mechanism of the reactions occurred during the experiment is explained in the supplementary information.

### Characterization

Crystal structure analysis was done with X-ray diffractometer (Rigaku MiniFlex) with Cu-Kα source radiation wavelength of 0.15418 nm. EDX, TEM and HRTEM characterizations were done on JEM-2100F by JEOL Ltd. For these characterizations SmCo_5_ and SmCo_5−x_ Cu_x_, samples were dispersed in hexane and the intermediate co-precipitation product samples were dispersed in ethanol. Ni TEM grid was used for TEM and HRTEM analyses. Magnetic properties (demagnetization curves) of SmCo_5_ and SmCo_5−x_Cu_x_ products were measured by Physical Property Measurement System (PPMS, Evercool II–9T) in the vibrating sample magnetometer mode.

## Results and discussion

**Figure 1:**
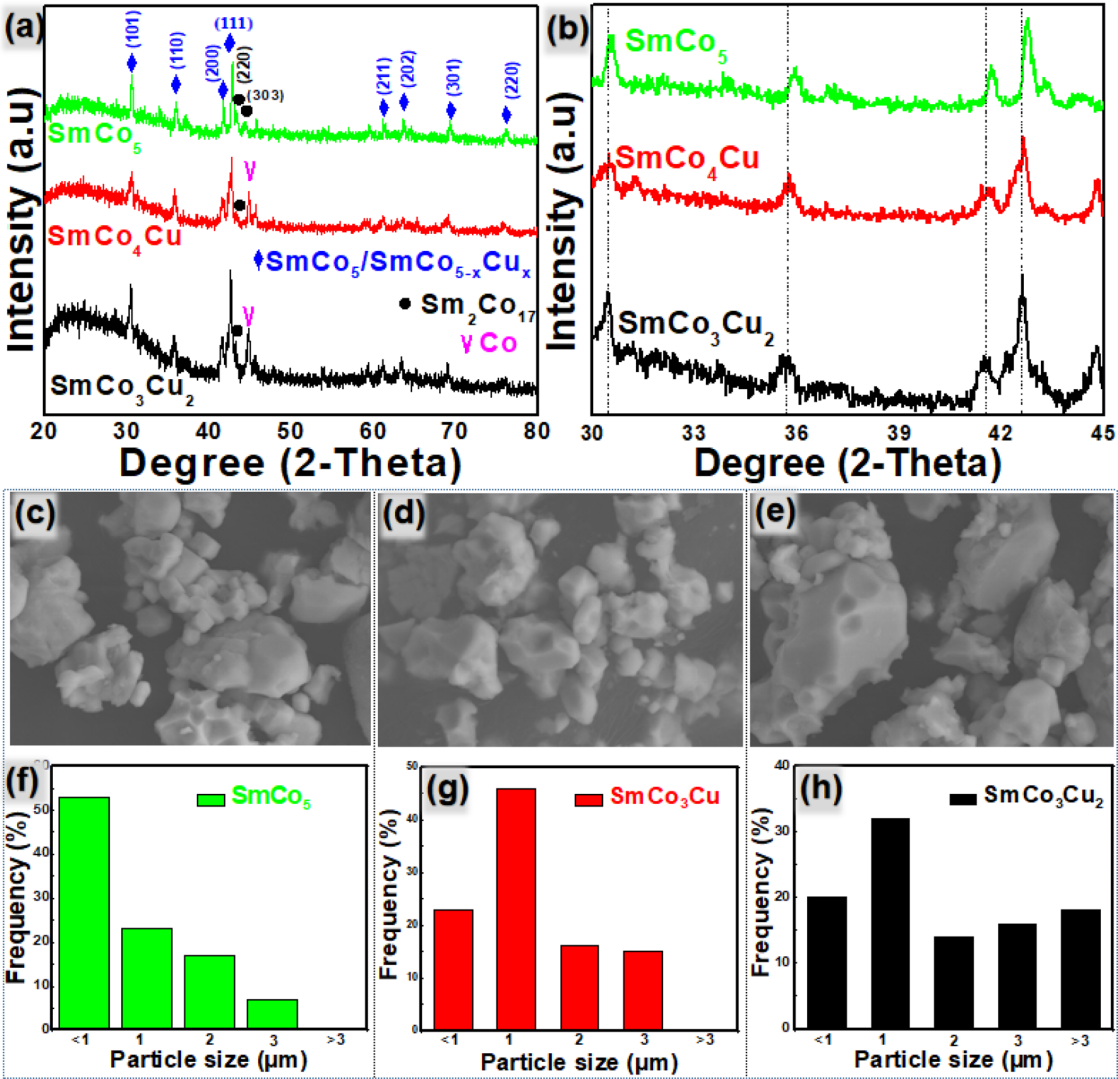
(**a**) XRD patterns of SmCo_5_, SmCo_4_Cu, and SmCo_3_Cu_2_. (**b**) Slight peak shift in XRD patterns of SmCo_5_, SmCo_4_Cu and SmCo_3_Cu_2._ SEM images of (**c**) SmCo_5_ (**d**) SmCo_4_Cu and (**e**) SmCo_3_Cu_2_. Particle size distribution of (**f**) SmCo_5_ (**g**) SmCo_4_Cu and (**h**) SmCo_3_Cu_2_.

SmCo_5_ and SmCo_5−x_Cu_x_ particles were produced by the experimental process described in the experimental section. XRD patterns for all SmCo_5_ and SmCo_5−x_Cu_x_ particles produced are similar (Fig. 1a) because the crystal structure of these products is quite similar. Co and Cu have nearly similar atomic radii (125 pm and 128 pm respectively) but these variations are detectable. Hence, there is slight enhancement in the d-spacing in the crystal of SmCo_5−x_Cu_x_ because of substitution of Co with Cu. This enhanced d spacing also shifts the peak to the smaller value of theta (θ) in XRD pattern (Fig. 1b). Cobalt peak is also observed around 45° (Fig. 1a) in SmCo_5−x_Cu_x._

SEM images in Fig. 1 reveal that the particles of all three products are in irregular shape and the size varies from 0.1 to 10 μm. Apparently it seems that Cu substitution enhances the particle size but exact mechanism behind this increased size is yet unknown. Maybe this due to faster reduction-diffusion in SmCo_5−x_Cu_x_ as compared to the SmCo_5._ Hence SmCo_5−x_Cu_x_ particles are formed earlier (as compared to the SmCo_5_) and stay for longer time at 900 °C. More annealing time can lead to the Ostwald ripening and resulted in increased size of particles.

**Figure 2:**
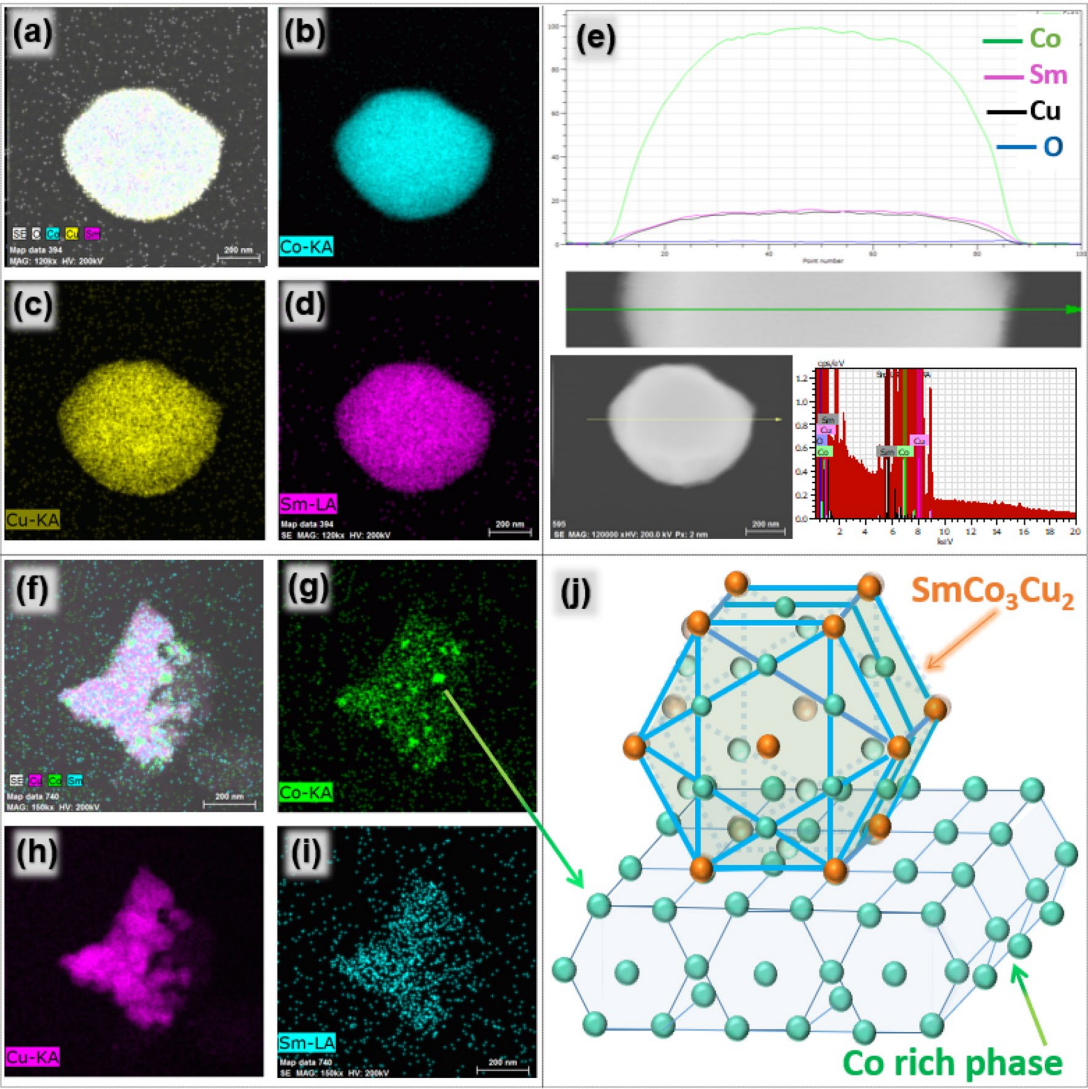
(**a**–**d**) TEM-EDS mapping images and (**e**) EDS line analysis of SmCo_4_Cu. (**f**–**i**) TEM-EDS mapping images SmCo_3_Cu_2_ and (**j**) schematic illustration of Co attachment on SmCo_3_Cu_2_.

Sm, Co and Cu are distributed evenly in the SmCo_5−x_Cu_x_ particles as shown in EDX mapping image of Fig. 2 that indicates the complete formation of SmCo_5−x_Cu_x_ alloy particles. TEM-EDS of SmCo_5_ is provided in supplementary information as Fig. S-7. Figure 2e is EDS line analysis of an isolated SmCo_4_Cu line. Line mapping confirms the homogeneous distribution of Cu throughout the particle. This also suggests the presence of Cu in the SmCo_4_Cu crystal, as elemental distribution is very regular. However, density of Sm, Co, and Cu is much higher in the center of the particle. This is because of higher thickness of the particle in the center. Small cobalt particles are attached on SmCo_3_Cu_2,_ can be detected in TEM-EDS images, Fig. 2f–i. Schematic illustration of SmCo_3_Cu_2_/Co interface is explained in Fig. 2j^25^.

**Figure 3:**
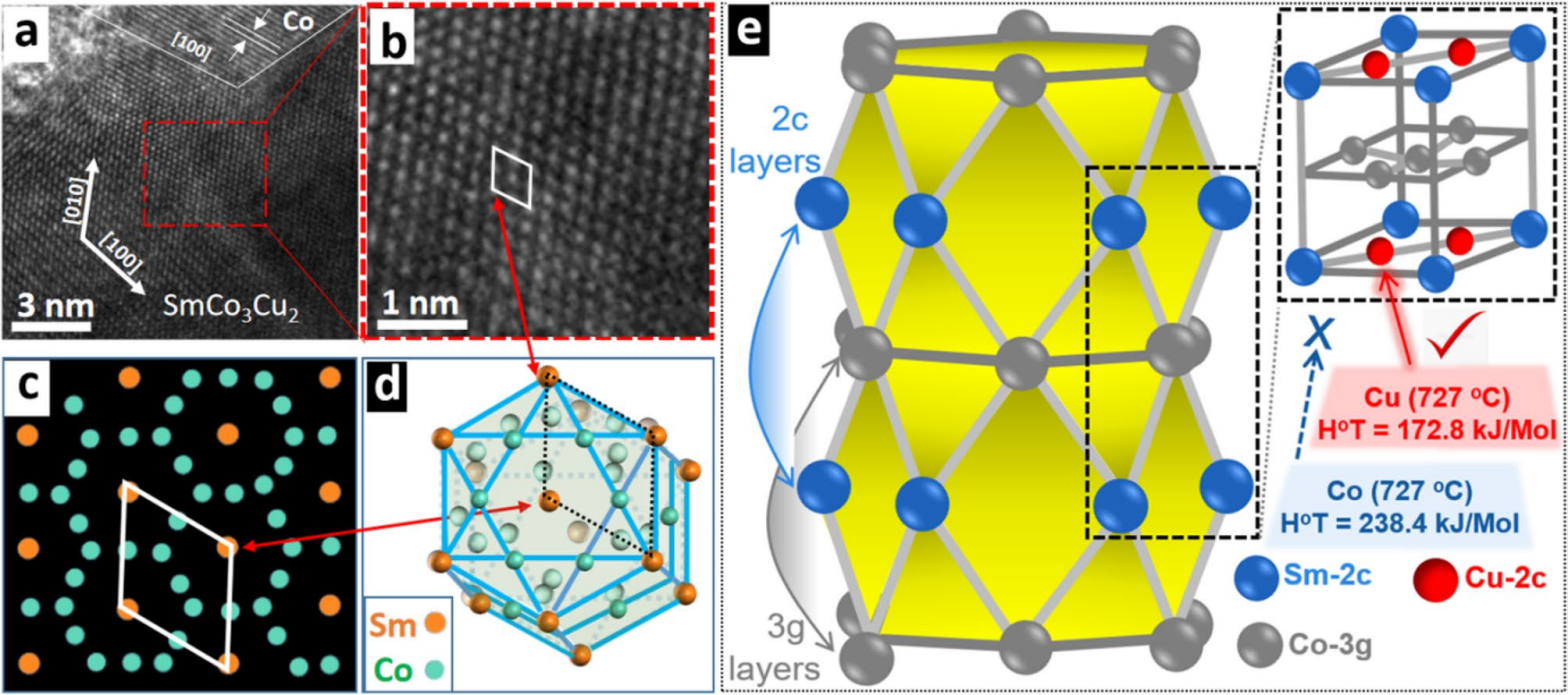
(**a**) HRTEM of SmCo_3_Cu_2_, (**b**) zoomed in area from the red dotted box in Fig. 2-a, (**c**) modeled SmCo_5_ structure, taken along “a/b” axis, (**d**) SmCo_5−x_Cu_x_/SmCo_5_ hcp crystal structure and (**e**) arrangements of atoms in the SmCo_5_ crystal lattice and the mechanism of Co substitution with Cu in “2c” layer of SmCo_5−x_Cu_x_ crystal lattice.

There is possibility that Co particles detected in TEM-EDS might be left after the reduction diffusion process and now exist as the free detached, Co particles in SmCo_4_Cu, and SmCo_3_Cu_2_. Other possibility is that these Co particles were fused with SmCo_4_Cu, and SmCo_3_Cu_2_ at high temperature during the reduction diffusion process, which was further proved by HRTEM analysis. Fused Co is identified at [100] facet in Fig. 3a. A portion of Fig. 3a, shown in red dotted box is zoomed in and described as Fig. 3b. Rhombus shown in Fig. 3d connects the four Sm atoms in the [001] zone axis. There are three Co atoms between these four Sm atoms but not visible in the TEM image because of their small size. Figure 3c is the modeling of SmCo_5_ structure which is taken along “a/b” axis showing four Sm atoms connected by rhombus^25^.

Figure 3d is the hexagonal cubic pack structure of the SmCo_5−x_Cu_x_/SmCo_5_. These four Sm atoms are arranged in the “a/b” axis of the crystal lattice. HRTEM analysis confirms the presence of Co and SmCo_3_Cu_2_ phases and their fusion in a single particle.

**Figure 4:**
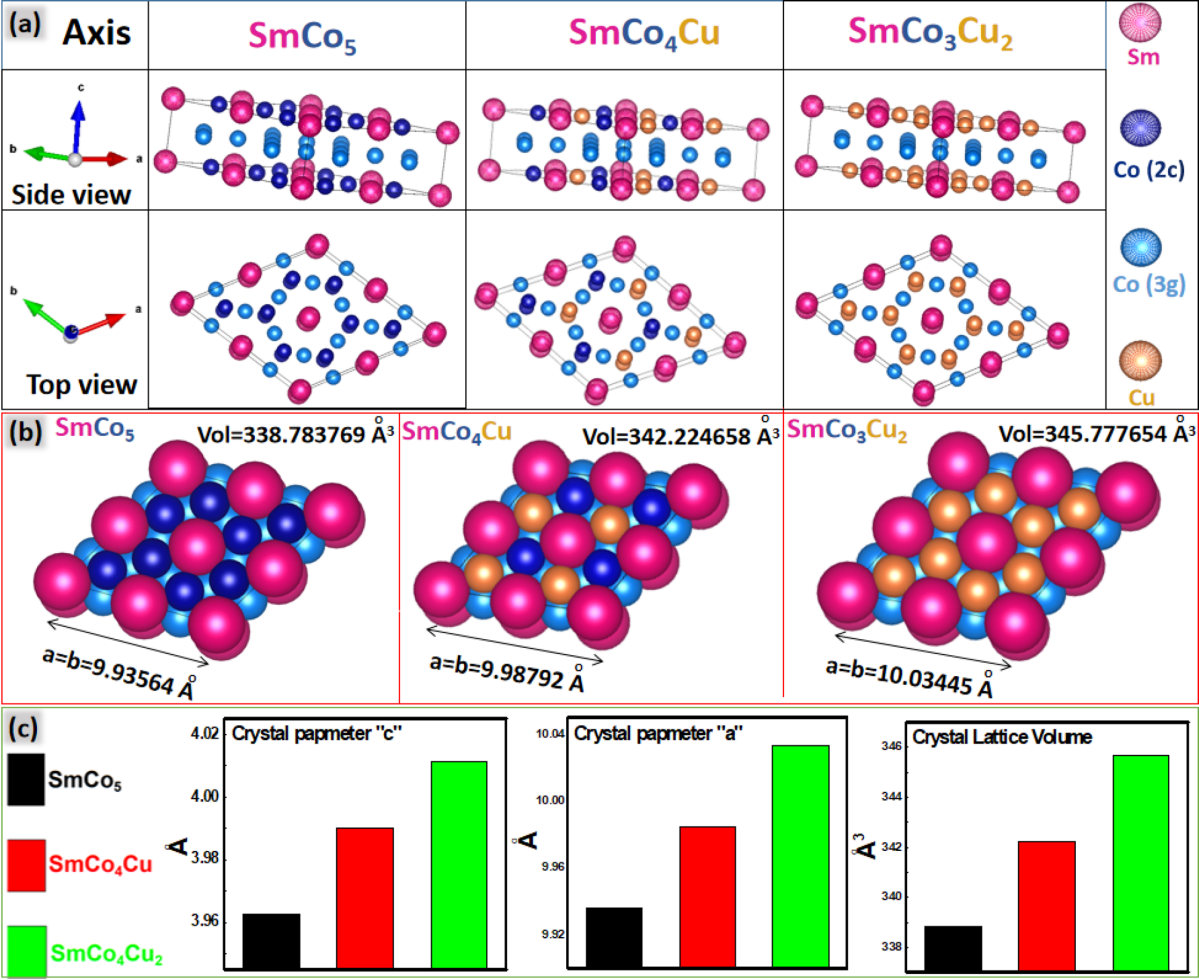
(**a**) Top and side view of arrangement of the atoms in SmCo_5_ and SmCo_5−x_Cu_x_, (**b**) space-filling model of SmCo_5_ and SmCo_5−x_Cu_x_ with crystal dimensions, (**c**) crystal parameters of SmCo_5_ and SmCo_5−x_Cu_x_.

During annealing at 900 °C, reduced oxides of Sm, Co and Cu were ready to diffuse and form new crystal. At 727 °C, Cu has lower enthalpy of the reduction (172.8 kJ/Mol) as compared to the Co (238.4 kJ/Mol)^26^. Hence Cu reduces and diffuses much faster than Co and all Cu becomes the part of the SmCo_5−x_Cu_x_ crystal lattice (Fig. 3e). Left over Co is found as fused Co phase, detected in XRD, HRTEM and EDS mapping. Sm and Co are distributed in the two layers of hcp crystal SmCo_5_, with the similar atomic distribution found in CaCu_5_ type crystals^27^. These layers are “3g” and “2c”. Co always occupies 3g layer. “2c” layer is shared among Co and Sm, where Sm are on the edges of the SmCo_5_ crystal and Co is located between the Sm atoms. Cu substitutes the Co in “2c” layer (Fig. 3e) and enhances the magneto-crystalline anisotropy^12^. Instability of Cu substitution at 3g sites is also confirmed by previous studies^12–28^. Cu substitution has been clearly explained (Fig. 4) with top and side view of arrangement of the atoms in SmCo_5_ and SmCo_5−x_Cu_x_.

Enhancement in the crystal parameters “a, b, c” and crystal volume was recorded after substitution. SmCo_5_ and SmCo_5−x_Cu_x_ have hexagonal close pack crystal structure. Crystal parameters in the hcp were calculated with the help of the following equation as reported by the Sinha et al.^29^

$$\frac{1}{{({\text{d}}_{{{\text{hkl}}}} )_{{{\text{hcp}}}}^{2} }} = \frac{4}{3}\left( {\frac{{{\text{h}}^{2} + {\text{hk}} + {\text{k}}^{2} }}{{{\text{a}}_{{{\text{hcp}}}}^{2} }}} \right) + \frac{{{\text{l}}^{2} }}{{{\text{c}}_{{{\text{hcp}}}}^{2} }}$$

“d” and “hkl” values were determined from the XRD patterns. Peak with [200] facet was selected and “d” spacing value for the [200] facet was determined from the XRD patterns. By putting the value of “d” in the equation above, value of the “a” parameter was calculated.

In the next step peak with the [111] facet was selected. Then “d” value for the [111] facet was determined and were put in the equation above. With the already known value of “a” parameter, value of “c” parameter was determined. In the hcp crystal “a = b”, hence no calculations for the “b” were performed. Substitution increased the unit cell volume by 3.9% (SmCo_4_Cu) and 5% (SmCo_3_Cu_2_) as shown in the Fig. 4. Collectively, all the crystal parameters were increased after the substitution.

**Figure 5:**
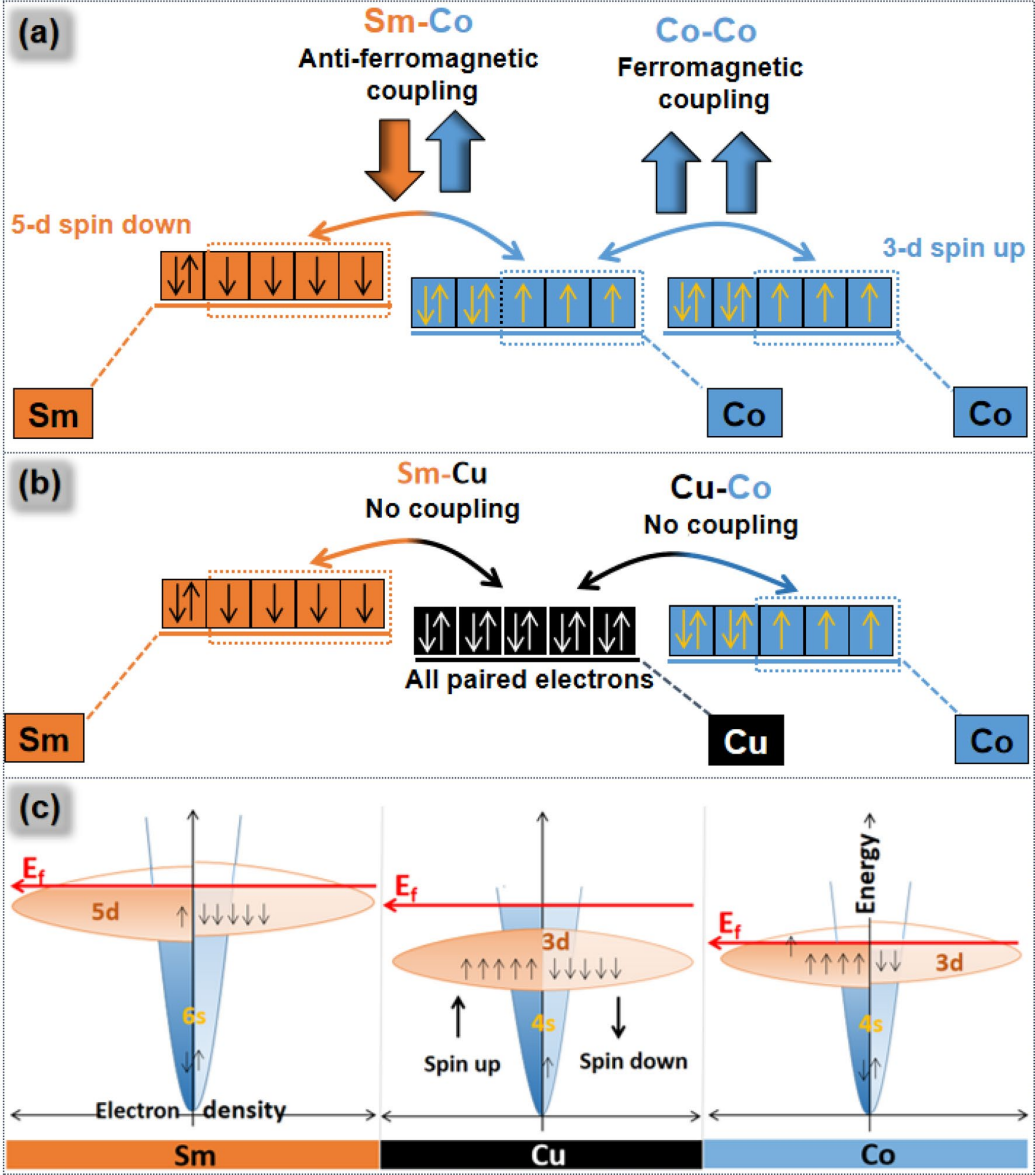
(**a**,**b**) Schematic illustration of exchange coupling between the electrons of Sm, Co and Cu, (**c**) rigid band model of Sm, Co and Cu.

With electronic configuration of [Xe] 4f^6^ 6s^2^, Sm has 6 unpaired electrons in 4f orbital. Before coupling with Co, electrons in the “f” orbital of Sm hybridize and move to the 5d orbital. Hence actual electronic configuration of the Sm in SmCo_5_ is [Xe] 5d^6^ 6s^2^ and it has four unpaired electrons in the valance shell. However, after the hybridization, 5d electrons are in the spin down state^30^. Valance unpaired electrons in Co are in spin up configuration, hence Sm couples antiferro-magnetically with the Co (Fig. 5a). Co with electronic configuration of [Ar] 3d^7^ 4s^2^ has three unpaired electrons in 3d orbital. Co has all the unpaired 3d electrons in the spin up configuration, which confirms that Co–Co exchange coupling is ferromagnetic (Fig. 5a).

Cu has electronic configuration of 3d^10^ 4s^1^ therefore it does not have any unpaired electron in the valance shell. Hence Cu being non-magnetic, does not couple with Co or Sm either (Fig. 5b).

When one Co, Cu or Sm atom come contact with the other Co, Cu or Sm atoms, their valence electrons make a valence band. This valence band can be explained with rigid band model (Fig. 5c). Density or abundance of the electrons is taken on X-axis while energy of electrons is taken on the Y-axis. E_f_ (fermi level) of the rigid band of the Cu is located in the “s” orbital which indicates that “3d” orbital is completely filled. It further tells that there is no inter-orbital movement of electrons in between 3d and 4s orbitals. All electrons in 3d orbitals are retained there unpaired therefore, being non-magnetic there is no chance for the Cu to couple with Sm or Co either. In case of Sm or Co, energy level of 3d electrons is similar to the 4s electrons, hence movement of electrons between the 4s and 3d orbital is possible. Fermi level of the Sm and Co is almost in the center of 3d band therefore, 3d band is partially filled (Fig. 4). Conclusively these band in Sm and Co are magnetic and can couple with each other.

**Figure 6:**
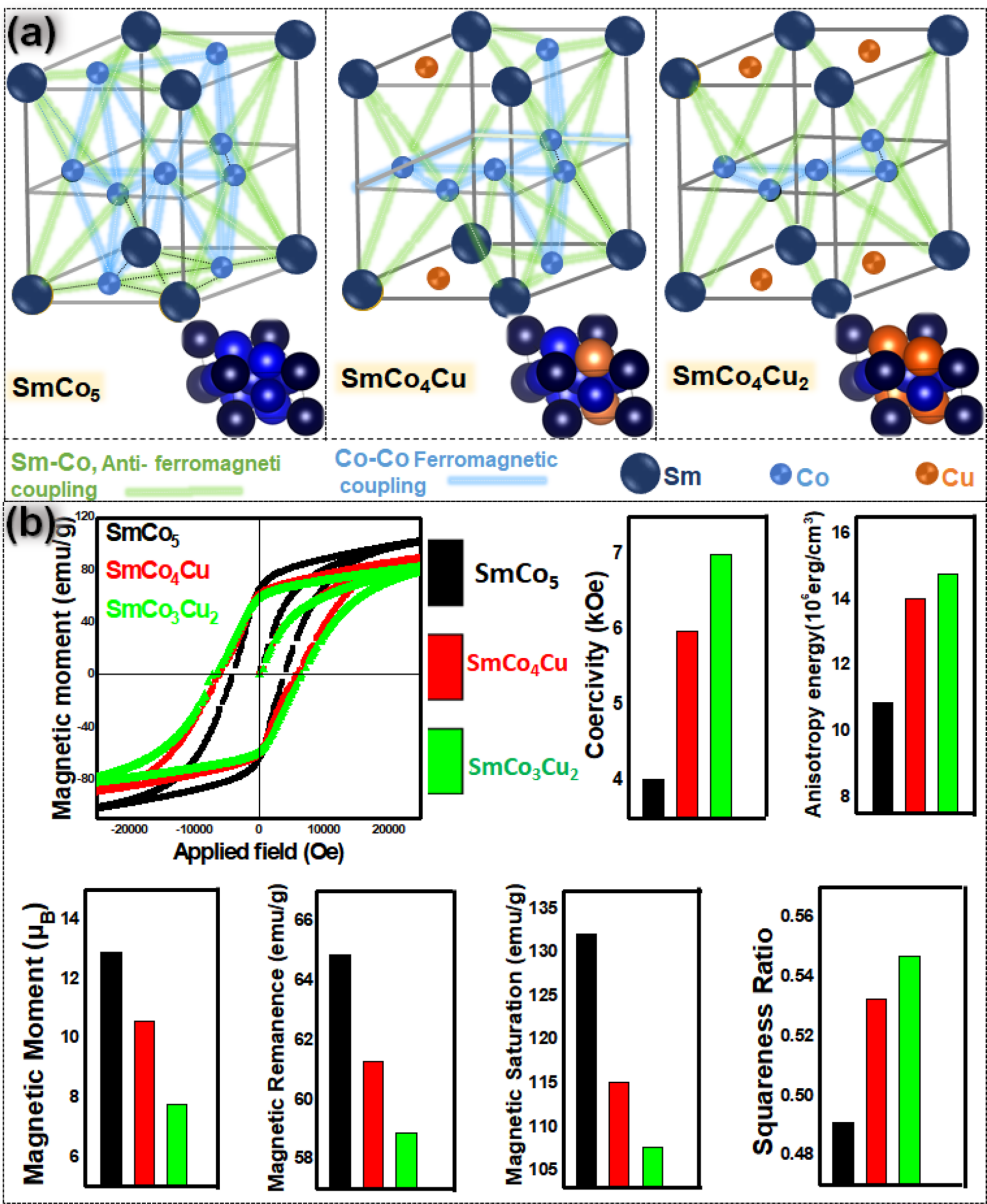
(**a**) Mechanism on the reduction of exchange coupling before and after Cu substitution in SmCo_5_ and SmCo_5−x_Cu_x_. (**b**) Magnetic hysteresis loops of SmCo_5_ and SmCo_5−x_Cu_x_. Variation of magnetic moment, H_c_, M_r_, M_s_ and anisotropy energy, before and after Cu substitution.

Cu reduced magnetic moment more effectively when it substitutes Co at “2c” position. Substitution at “3g” site can eliminate the anisotropy energy, in consequence magnetic properties may vanish^12^. SmCo_4_Cu or SmCo_3_Cu_2_ are anisotropic and easily magnetized from the a/b dimension of the crystal lattice (Fig. 6a). “2c” site substitution can decouple the ferromagnetic interaction between cobalt atoms of “2c” and “3g” layers and magnetic moment between these layers reduces drastically. Hence magnetization in the a/b direction (easy direction for magnetization) of the crystal becomes harder, that leads to the enhancement of coercivity and reduction of Mr value. It is concluded that in this work substitution occurred at the “2c” site as shown in the Fig. 6a.

Being non-magnetic after substitution, Cu strongly affects the magnetic properties of SmCo_5._ Measured magnetic hysteresis curves for the SmCo_5−x_Cu_x_ and SmCo_5−x_Cu_x_ are shown in Fig. 6b. The magnetic moment of SmCo_5_, SmCo_4_Cu and SmCo_3_Cu_2_ are to be 12.86, 10.58 and 7.79 μ_B_, respectively. In our work magnetic moment for SmCo_5_ and SmCo_5−x_Cu_x_ was calculated from the M_s_ values in the hysteresis loop. These values are lower than theoretically calculated values, because the theoretical conditions are ideal conditions (e.g. no thermal energy in the system and no oxidation of the product). The measured hysteresis curves for the SmCo_5_ and SmCo_5−x_Cu_x_ are shown in Fig. 6b.

Reduction in magnetic moment critically affected on the anisotropy energy and coercivity. Coercivity (Hc) values of SmCo_5_, SmCo_4_Cu and SmCo_3_Cu_2_ were recorded as 4.5, 5.97 and 6.99 kOe, respectively. The increasing order of coercivity of all three products was determined as SmCo_5_ < SmCo_4_Cu < SmCo_3_Cu_2_. Simultaneously decreasing order of magnetic moment is SmCo_5_ > SmCo_4_Cu > SmCo_3_Cu_2_.

Energy density or energy product is intrinsic property and is the amount of energy stored in the lattice because of arrangement of the atoms in the crystal. It was found that Cu substitution enhanced the energy density. Energy densities for SmCo_5_, SmCo_4_Cu and SmCo_3_Cu_2_ were recorded as 10.87, 14.05, and 14.78 M erg/cm^3^ respectively. Furthermore, it is also evident from the hysteresis loop that squareness ratio was also increased after the Cu substitution. Complete hysteresis loop is given in the supplementary information as Fig. S-8. Possible effect of Cu substitution on the domain wall of SmCo_5−x_Cu_x_ is also explained in the supplementary information.

Role of the extra Co phase present in the SmCo_5−x_Cu_x_ is very important. Cu enhances the coercivity at the cost of magnetic moment. Formation of SmCo_3_Cu_2_ after the substitution of Cu in SmCo_5_ can reduce the M_r_ value up to 35%^12^ and 32%^31^. Reduced M_r_ value will reduce the energy density drastically despite of enhancement of the coercivity. Purpose of keeping the extra Co phase was to maintain the M_r_ value as well as the cost reduction of the product. Proposed mechanism of reduction of M_r_ value by coupling between Co and SmCo_3_Cu_2_ is explained in Fig. S-9.

Coercivity of the SmCo_5_ particles produced by chemical method is quite low as compared to the SmCo_5_ magnets. But the coercivity reported in our work is still relatively lower as compared to the coercivity reported in other recent chemical methods. One reason behind the lower value of the coercivity is the presence of small Sm_2_Co_17_ phase (Fig. 1). Second main reason for the lower coercivity value is the irregular size and morphology of the magnetic particles. It is common observation, that when particle size of magnetic powder approaches the single domain size, magnetic properties are excellent. Similar conclusion was concluded by the Ma et al.^5^ and they provided the magnetic properties of the mono-dispersed particles. Chuev et al. and Chen et al. explained the size and morphology dependence of magnetic properties (especially coercivity in detail)^32,33^. In our work, particles with the similar size (average size 300 nm) were also separated and they exhibited much better magnetic properties. Coercivity of SmCo_3_Cu_2_ was recorded as 13.5 kOe. Detail of this experiment and results obtained is described in the supplementary information.

## Conclusion

SmCo5 and SmCo_5−x_Cu_x_ magnetic particles were synthesized by energy efficient, chemical method. Microstructure confirmed the Cu substitution in the SmCo_5_ lattice and presence of Co phase fused together with SmCo_5−x_Cu_x_. After substitution at “2c” site in the SmCo_5_ crystal lattice, Cu almost blocked the coupling in the surrounding. The resulted decoupling in the crystal lattice affected the magnetic moment, anisotropy and coercivity. Magnetic moment was reduced as the result of Cu substitution, but coercivity and anisotropy energy were enhanced. The substitution of Co with Cu and extra Co phase decreased the price of SmCo_5−x_Cu_x_, up to 5%.

## Supplementary Information

Supplementary Information.
