# Supplementary material for: Enhancement of anisotropy energy of SmCo5 by ceasing the coupling at 2c sites in the crystal lattice with Cu substitution
Source: Sci Rep. 2021 May 12;11:10063. doi: 10.1038/s41598-021-89331-z (PMC8115628; doi:10.1038/s41598-021-89331-z)
Supplement: Supplementary file 1 — Supplementary Information. [file 41598_2021_89331_MOESM1_ESM.docx]

Enhancement of Anisotropy Energy of SmCo_5_ by Ceasing the Coupling at 2c Sites in the Crystal Lattice with Cu Substitution

*Syed Kamran Haider^a,b,c^, Hieu Minh Ngo^a^, Dongsoo Kim^b,c^, Young Soo Kang^a^**

^a^ Department of Chemistry, Sogang University, 35, Baekbeomro, Mapogu, Seoul, 04107, South Korea.

^b^ Powder & Ceramics Division, Korea Institute of Materials Science, Changwon, Gyeongnam 51508, South Korea.

^c^ Convergence research center for development of mineral resources, Korea Institute of Geoscience and Mineral Resources, Daejeon 34132, South Korea.

*Corresponding author.

Email: [yskang@sogang.ac.kr](mailto:yskang@sogang.ac.kr)

Telephone: +82-2-702-6379

**Chemical reactions in the experiment**

Possible chemical reactions (following the scheme proposed by Ma et. al^1^) in our process are given below. In first step NaOH converts Sm, Co and Cu chloride compounds to their hydroxides as the following reactions.

SmCl_3_ ＋ 3NaOH → Sm(OH)_3_ ↓＋ 3NaCl

CoCl_2_ ＋ 2NaOH → Co(OH)_2_ ↓＋ 2NaCl

CuCl_2_ ＋ 2NaOH → Cu(OH)_2_↓ ＋ 2NaCl

Hydroxides change to the oxides during heating in CVD furnace. Ca added into the metal oxides reduces them to metallic form, Sm, Co and Cu atoms fuse together to form SmCo_5-x_Cu_x_ alloy.

CoO ＋ Ca → Co ＋ CaO

CuO ＋ Ca → Cu ＋ CaO

Sm_2_O_3_ ＋ 3Ca → 2Sm + 3CaO

*x*Cu ＋ (5-*x*)Co ＋ Sm → SmCo_5-_*_x_*Cu*_x_*

**Enhancement of anisotropy by domain wall kink formation**

Domain wall energy γ increases with increasing Co substitution with Cu in SmCo_5_ crystal lattice, which increases the coercivity.^2^ When Co was substituted with Cu in SmCo_5_, Cu increases its crystallization^3^ and increases its anisotropy field.^4^ Addition of Cu into SmCo_5_ affects the domains walls by formation of domain wall kinks. Domain wall kinks are the local deformations in the domain wall. These kinks are responsible for the difference in magnetic properties between SmCo_5_ and SmCo_5-x_Cu_x_.^5^ Furthermore, domain wall kink width depends indirectly on the magnetic anisotropy constant, K_1_ that increases with the Cu substitution in SmCo_5_. Domain wall kink depends on Bloch domain wall energy є that depends on anisotropy constant K_1._^6^

Є = 4$\sqrt{\text{AK}_{1}}$

**Sources of costs and of relative abundance the Sm, Co and Cu**

Prices for all the metals and alloys shown in Fig. S-1 are taken on the December 31^st^ of each year and details of the source of the data is provided in the below.

1-Link for price of Cu. (<https://www.macrotrends.net/1476/copper-prices-historical-chart-data>)

2-Link for price of Co (<https://www.researchgate.net/publication/326353145_Prospects_for_electric_vehicle_batteries_in_a_circular_economy/link/5b48685445851519b4b4ef7b/download>).

3-Link for price of Sm.(<https://www.amazon.com/Samarium-metal-99-95-pure-20g/dp/B01MT6T3WR>)

Link for price of relative abundance of Sm Co Cu in earth crust. (<https://periodictable.com/Properties/A/CrustAbundance.an.log.html>)

4-Link for Past and expected future market share of SmCo_5_ in global market. (<https://www.waltbenecki.com/uploads/more_than_you_ever_wanted_to_know.pdf>)

Figures and Figure Captions


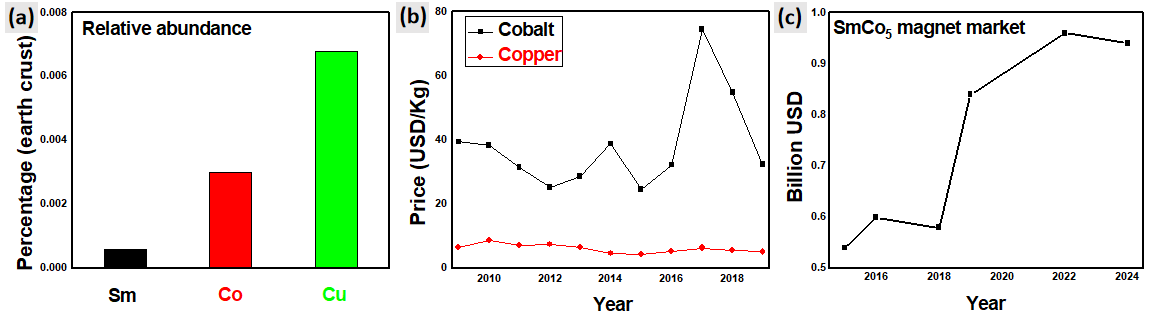


FigureS-1. (a) Relative abundance of Sm, Co and Cu elements in the earth crust, (b) price comparison of Co and Cu during last decade and (c) past and expected future market share of SmCo_5_ in global market.


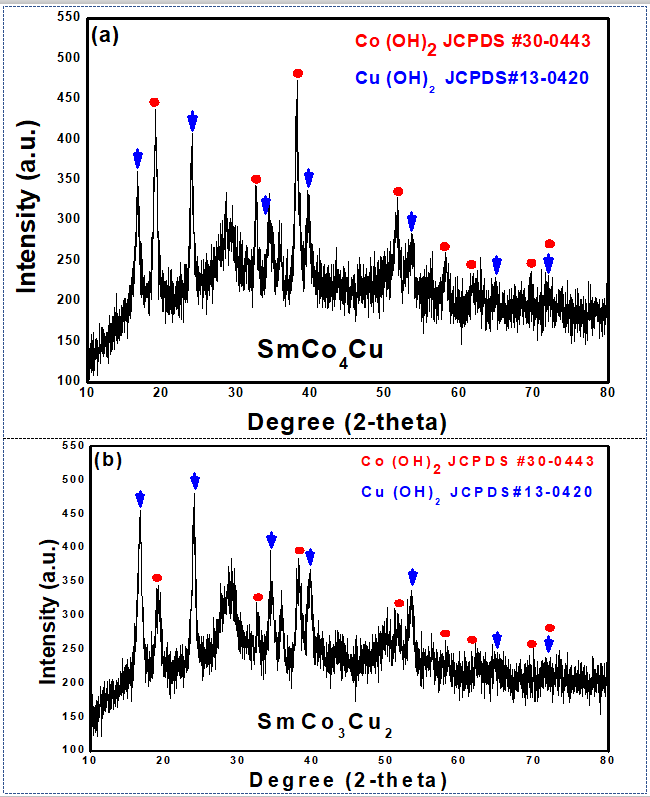


Figure 2. XRD patterns of hydroxide intermediates of (a) SmCo_4_Cu, and (b) SmCo_3_Cu_2_. In XRD peaks for Sm(OH)_3_ are not detectable, because it is amorphous, although a broad peak around 30 degree of 2-theta can be Sm(OH)_3._


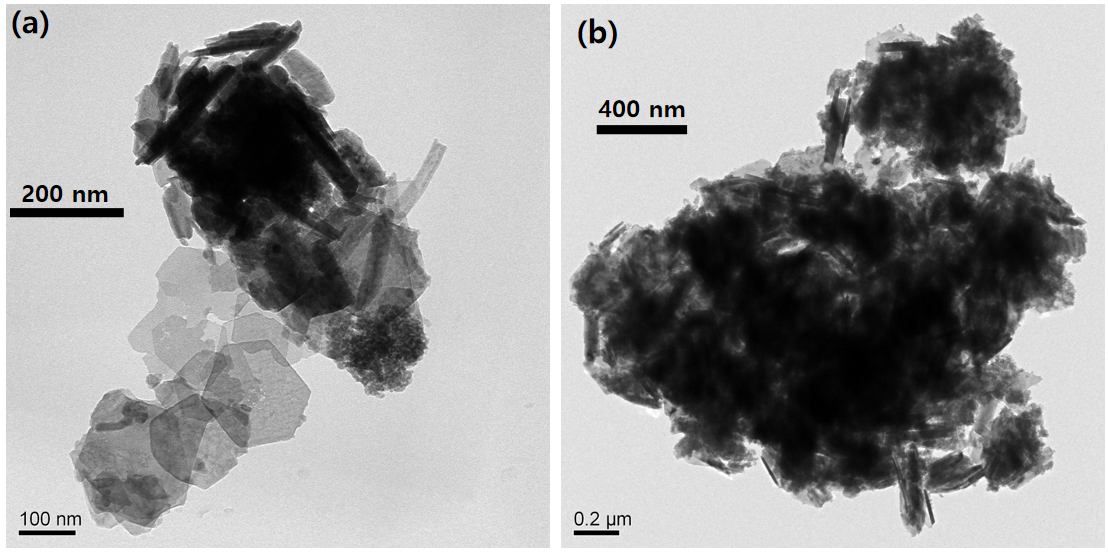


Figure 3. TEM images for hydroxide intermediates of (a) SmCo_4_Cu and (b) SmCo_3_Cu_2_.


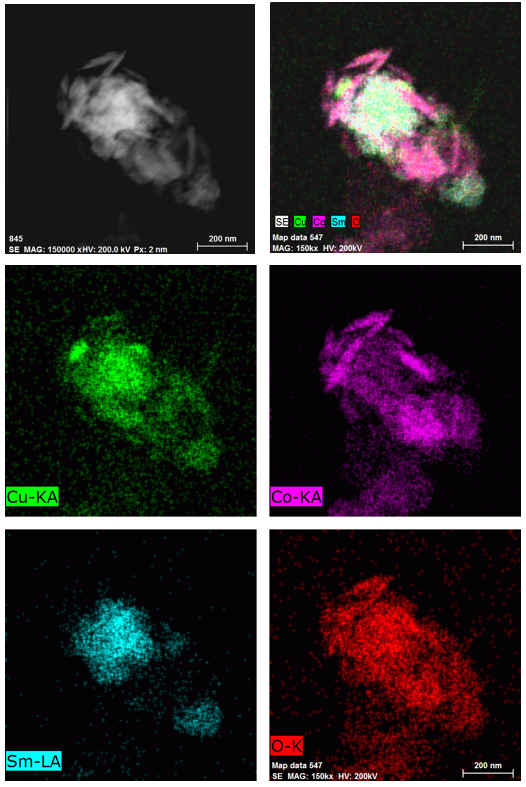


Figure 4. TEM-EDS mapping images for hydroxide intermediates of SmCo_4_Cu.


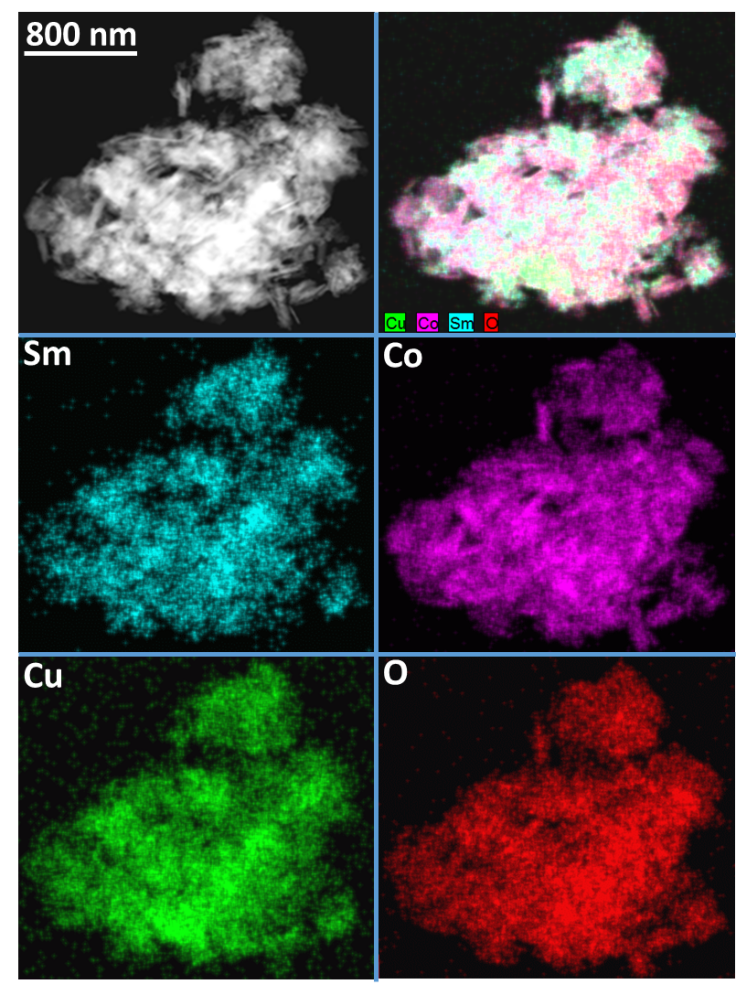


Figure 5. TEM-EDS mapping images for hydroxide intermediates of SmCo_3_Cu_2_.


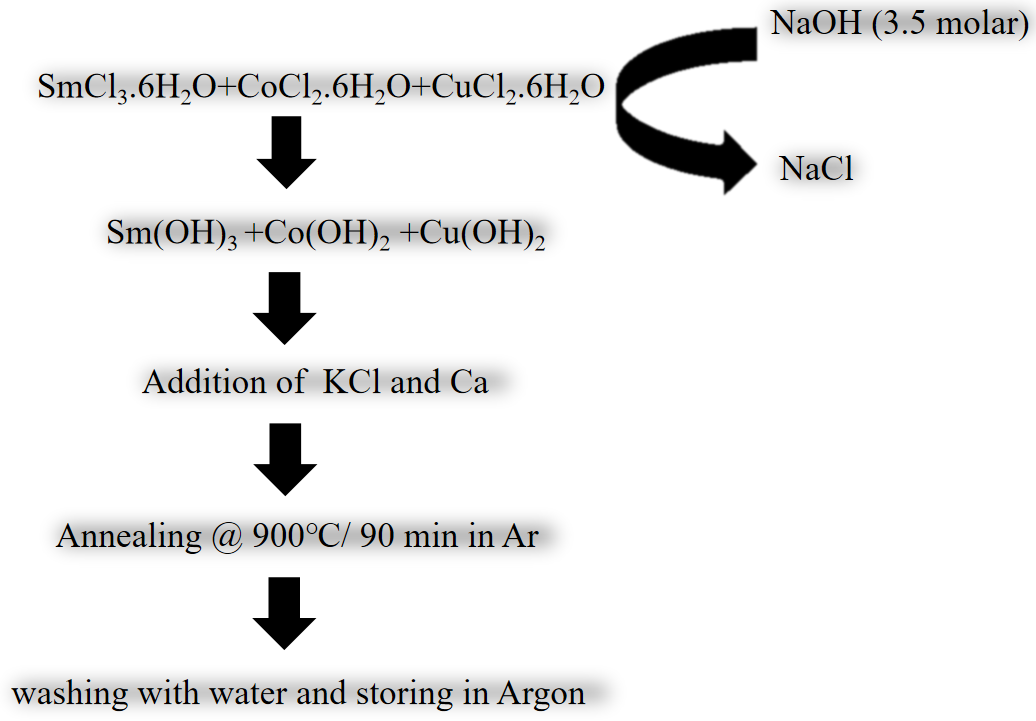


Figure S-6 Experimental process for the synthesis of SmCo_5_, SmCo_4_Cu, and SmCo_3_Cu_2_.


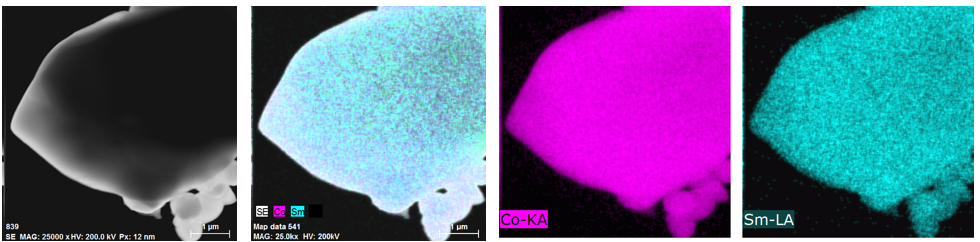


Figure S-7. TEM-EDS images of SmCo_5_.


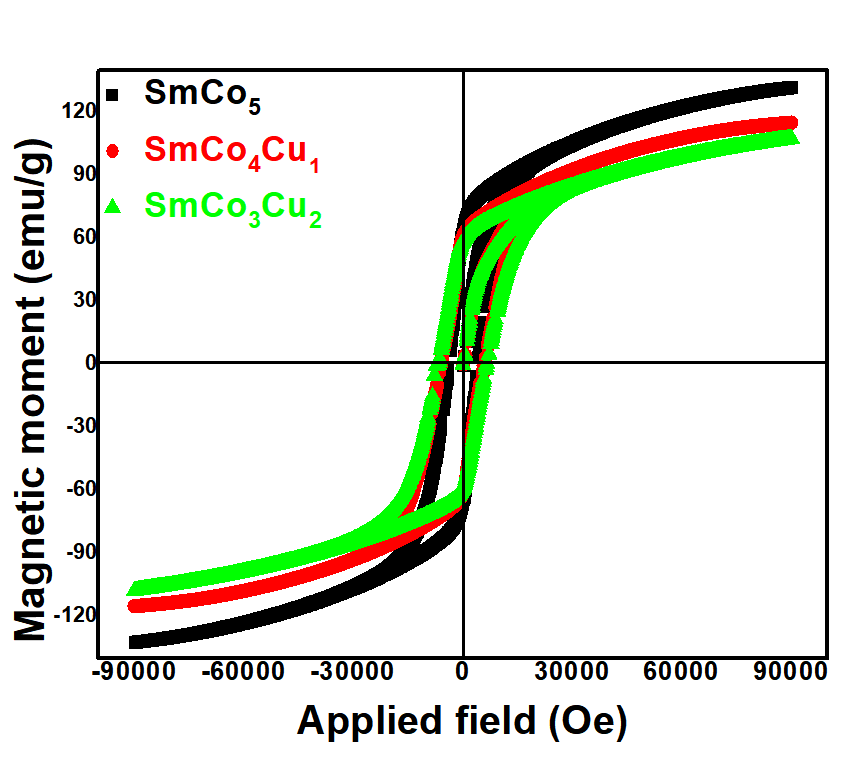


Figure S-8. Magnetic hysteresis loops of SmCo_5_ and SmCo_5-x_Cu_x ._


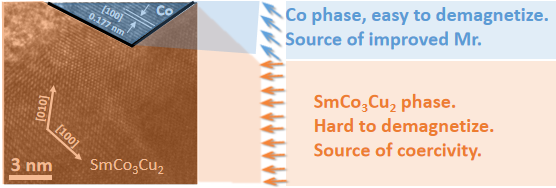


FigureS-9. Exchange interaction between SmCo_3_Cu_2_ and Co.

**Isolation of smaller particles and their characterization**

To obtain the particles with homogeneous size and morphology at first magnetic powder was ultra-sonicated resulting the suspension of the very small particles in the solvent (water). These small particles with size less than ~100 nm were separated by repeated ultra-sonication and removal of the solvent. Next target was to separate the particles with the size of less than 500 nm. In order to do it, filter paper with pore size of 500 nm was used. Smaller particles of SmCo_5_, SmCo_4_Cu and SmCo_3_Cu_2_ were obtained after filtration. SEM images and magnetic properties of the isolated SmCo_5_ and SmCo_5−x_Cu_x_ particles are given below (Fig. S-10).


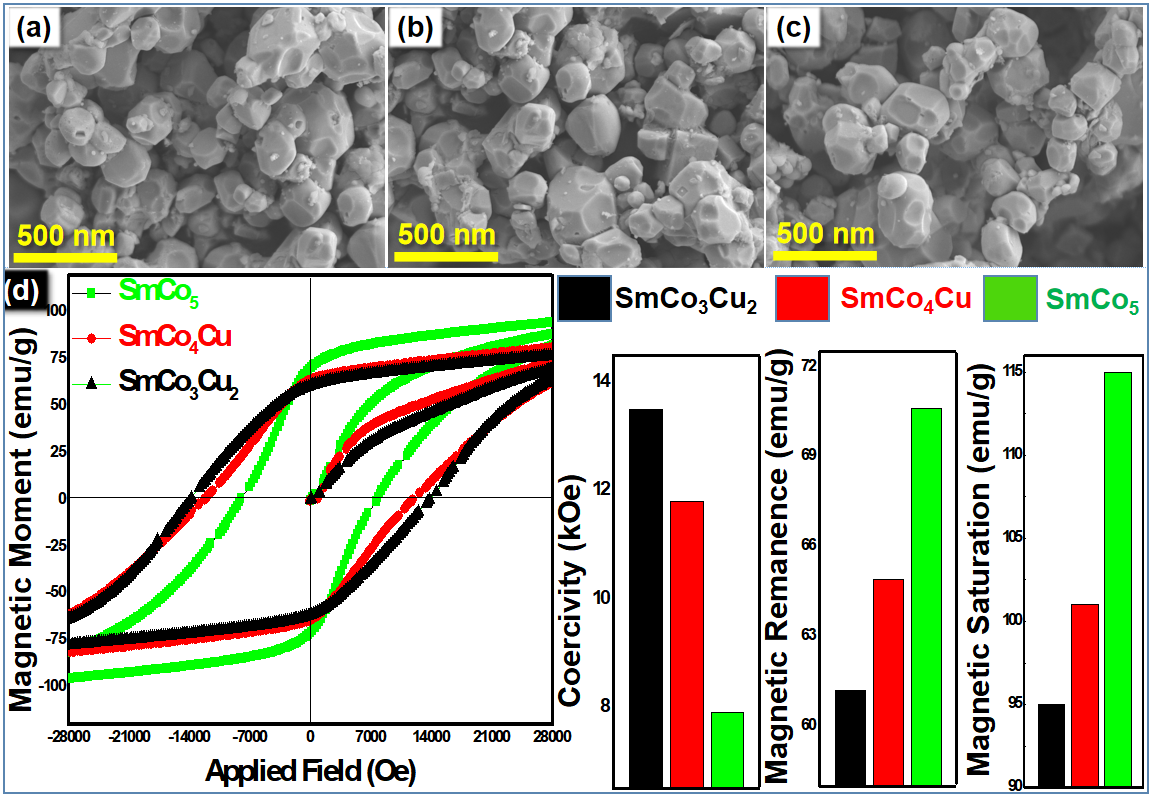


Fig. S-10. SEM images of (a) SmCo_5_ (b) SmCo_4_Cu (c) SmCo_3_Cu_2_ and (d) magnetic properties of SmCo_5_ and SmCo_5−x_Cu_x_.

References

1. Ma, Z., Zhang, T. & Jiang, C. A facile synthesis of high performance SmCo5 nanoparticles. *Chem. Engg. J*. **264,** 610–616 (2015). <https://doi.org/10.1016/j.cej.2014.11.138>

[2] Oesterreicher, H. On the coercivity of cellular Sm_2_Co_17_-SmCo_5_ permanent magnets. *J. Less-Common Met*. **99,** L17 - L20 (1984).

[3] Ohtake, M., Nukaga, Y., Kirino, F. & Futamoto, M. Effects of substrate temperature and Cu underlayer thickness on the formation of SmCo_5_ (0001) epitaxial thin films. *J. Appl. Phys*. **107,** 09A706, 01-03 (2010).  <https://doi.org/10.1063/1.3334539>

# [4] Lectard, E., Allibert, C.H. & Ballou, R. Saturation magnetization and anisotropy fields in the Sm(Co_1−_*_x_*Cu*_x_*)_5_ phases. *J. Appl. Phys.* 75, 6277 (1994). <https://doi.org/10.1063/1.355423>

[5] Uehara, M. & Barbara, B. Noncoherent quantum effects in the magnetization reversal of a chemically disordered magnet: SmCo_3.5_Cu_1.5_ *J. Phys. France.* **47**, 235-238 (1986). DOI: [10.1051/jphys:01986004702023500](https://www.researchgate.net/deref/http://dx.doi.org/10.1051/jphys:01986004702023500)

[6] Orval, N. Master research project. Institute for Molecules and Materials, Radboud University July 15(2015).
